# Editorial: Highlights in psychology of aging: impact of the COVID-19 pandemic on older adults

**DOI:** 10.3389/fpsyg.2023.1326725

**Published:** 2023-11-14

**Authors:** Izolde Bouloukaki, Tianyuan Li, Gianpaolo Maggi, Alessia Rosi

**Affiliations:** ^1^Department of Social Medicine, School of Medicine, University of Crete, Heraklion, Greece; ^2^Sleep Disorders Center, Department of Respiratory Medicine, School of Medicine, University of Crete, Heraklion, Greece; ^3^Division of Applied Psychology, School of Humanities and Social Science, The Chinese University of Hong Kong, Shenzhen, Shenzhen, China; ^4^Department of Psychology, University of Campania “Luigi Vanvitelli”, Caserta, Italy; ^5^Department of Brain and Behavioral Sciences, University of Pavia, Pavia, Italy

**Keywords:** COVID-19, older adults, sleep disorders, mental health, physical health

The COVID-19 pandemic has had a profound impact on the lives of many individuals globally. Older adults, specifically, were deemed more susceptible to the infection and its consequences when compared to their younger counterparts. The lockdown disrupted their daily routines, overwhelmed hospitals made it difficult to access timely healthcare services, the economic recession resulted in financial loss, mass quarantine led to separation from family members, and limited internet access and lack of smartphones posed difficulties in using digital technologies (Yang et al., 2020; Zhong et al., 2020; Weil et al., 2021). Indeed, soon after the outbreak, it was identified that age is a determining factor for both mortality rates and susceptibility to virus transmission (Bonanad et al., 2020; Davies et al., 2020).

Despite high vulnerability to the COVID-19 infection, older adults report higher resilience and better psychological adjustment compared to their younger counterparts during the pandemic. In particular, older age is related to less anxiety and depression (Bruine de Bruin, 2021), higher positive affect, lower negative affect, and lower reactivity to negative events facing the pandemic (Klaiber et al., 2021). Reasons for such age differences are multifaceted. Proactive coping strategies may aid older adults in adjusting to the pandemic threat (Pearman et al., 2021), while young adults' psychological wellbeing suffers more due to the impact of the pandemic and quarantine measures on their daily lives and social relationships (Birditt et al., 2021; Klaiber et al., 2021). Best et al. in the current topic, conducted a 29-wave survey and found that older adults consistently experienced less psychological distress over the entire 16-month pandemic period. The presence of anxiety and depression diagnoses did not affect the decrease in age differences over time. Similarly, Serrao Hill et al. examined the correlation between mental health symptoms and happiness in older adults, both before and after the pandemic was declared. They found that older adults who reported higher levels of anxiety and depressive symptoms showed higher happiness levels after the pandemic, compared to their pre-pandemic levels of happiness. Moreover, Fu et al. demonstrated that even older adults who are at High Risk of Cognitive Impairment (HRCI) were able to emotionally cope with the negative stressors brought on by the pandemic. In fact, these individuals exhibited a reduction in negative emotional states during the remission period as compared to the outbreak period, indicating their ability to manage the pandemic-related stressors. These studies suggest that older adults' resilience and experience with challenging life events have helped them develop effective coping strategies to better manage stressors during the pandemic (Lind et al., 2021; Rapisarda et al., 2023).

The presence of social support networks seems also to contribute to older adults' emotional wellbeing during the pandemic (Cavallini et al., 2021). Jiang and Carstensen found that older adults preferred to interact with emotionally close social partners over novel social partners both during the peak of the pandemic and after vaccines had become available. Serrao Hill et al. found that having a close social partner contributed to higher levels of subjective wellbeing among older adults. These findings suggest that a close partner can promote older adults' wellbeing, even in a stressful situation. Conversely, according to Panes Lundmark et al. low social network satisfaction emerged as a key predictor of persistent loneliness among older adults both prior to and during the pandemic. Of note, Sams et al. found that a substantial 83% of older adults were concerned about the disruption of their social activities as a consequence of the social restrictions implemented during the COVID-19 pandemic. Overall, the findings of these studies highlight the importance of supporting older adults in maintaining meaningful social engagement as a mean of managing stressful situations, including those associated with the pandemic.

Considering that in older adults there is a natural decline in cognitive functioning, it is conceivable to hypothesize that long period of quarantine may indirectly lead to feelings of reduced cognitive efficiency due to disruptions in daily routines (Fiorenzato et al., 2021; Maggi et al., 2021, 2022; Santangelo et al., 2021). Interestingly, Panes Lundmark et al. revealed that, although cognitive impairment did not emerge as significant risk factor for situational and persistent loneliness, cognitive impairment was more frequent within individuals living in persistent loneliness than individuals that did not experience loneliness. Conversely, Fu et al. did not reveal an influence of participants' behavioral and emotional coping skills on cognition. Sutton et al. examined whether poorer psychological and physical wellbeing was associated with worries about cognitive functioning and concerns about developing dementia in older adults during the COVID-19 pandemic. These authors revealed that 25.1% of participants reported concerns about their cognitive health, and that these worries were significantly related not only to poorer psychological wellbeing but also to worse physical conditions in terms of fatigue and poorer sleep quality and quality of life.

It is worth pointing out that both subjective cognitive decline (Tsapanou et al., 2019) and cognitive impairment (Wennberg et al., 2017) appear to be associated with sleep problems in older adults. The above relationships mentioned above may become more pronounced during times of extreme hardship, like the COVID-19 pandemic. Based on data from 914 elderly individuals, Almonds et al. observed that 71% of participants had a pre-existing sleep disorder, and certain conditions worsened during the pandemic, notably insomnia in women and obstructive sleep apnea in men. Individuals with primary education displayed higher sleep latency in comparison to those with a Ph.D., indicating an influence of educational level on sleep latency. In addition, disturbances in sleep were correlated with symptoms of depression, anxiety, and irritability in older individuals. It is plausible to suggest that during a difficult period, such as the COVID-19 pandemic, the practice of social distancing and isolation may further intensify existing sleep problems and psychological distress in the older population, ultimately resulting in more adverse health status outcomes (Lebrasseur et al., 2021).

In summary, the articles included in this Research Topic provided us with valuable insights into the consequences of the COVID-19 pandemic on the overall wellbeing of older adults, and also offered potential strategies to enhance the physical and mental wellbeing of the aging population in the aftermath of catastrophic events such as the COVID-19 pandemic.

## Author contributions

IB: Writing—original draft, Writing—review & editing. TL: Writing—original draft, Writing—review & editing. GM: Writing—original draft, Writing—review & editing. AR: Writing—original draft, Writing—review & editing.

## References

[B1] Birditt K. S. Turkelson A. Fingerman K. L. Polenick C. A. Oya A. (2021). Age differences in stress, life changes, and social ties during the covid-19 pandemic: implications for psychological well-being. Gerontologist 61, 205–216. 10.1093/geront/gnaa204 33346806 PMC7799124

[B2] Bonanad C. García-Blas S. Tarazona-Santabalbina F. Sanchis J. Bertomeu-González V. Fácila L.. (2020). The effect of age on mortality in patients with COVID-19: a meta-analysis with 611,583 subjects. J. Am. Med. Direct. Assoc. 21, 915–918. 10.1016/j.jamda.2020.05.045 32674819 PMC7247470

[B3] Bruine de Bruin W. (2021). Age differences in COVID-19 risk perceptions and mental health: evidence from a national U.S. survey conducted in March 2020. J. Gerontol. 76, e24–e29. 10.1093/geronb/gbaa074 32470120 PMC7542924

[B4] Cavallini E. Rosi A. van Vugt F. T. Ceccato I. Rapisarda F. Vallarino M.. (2021). Closeness to friends explains age differences in positive emotional experience during the lockdown period of COVID-19 pandemic. Aging Clin. Exper. Res. 33, 2623–2631. 10.1007/s40520-021-01927-7 34247344 PMC8272682

[B5] Davies N. G. Klepac P. Liu Y. Prem K. Jit M. CMMID COVID-19 working group Eggo, R. M. (2020). Age-dependent effects in the transmission and control of COVID-19 epidemics. Nat. Med. 26, 1205–1211. 10.1038/s41591-020-0962-9 32546824

[B6] Fiorenzato E. Zabberoni S. Costa A. Cona G. (2021). Cognitive and mental health changes and their vulnerability factors related to COVID-19 lockdown in Italy. PLoS ONE 16, e0246204. 10.1371/journal.pone.0246204 33503055 PMC7840042

[B7] Klaiber P. Wen J. H. DeLongis A. Sin N. L. (2021). The ups and downs of daily life during COVID-19: Age differences in affect, stress, and positive events. J. Gerontol. 76, e30–e37. 10.1093/geronb/gbaa096 32674138 PMC7454856

[B8] Lebrasseur A. Fortin-Bédard N. Lettre J. Raymond E. Bussières E. L. Lapierre N.. (2021). Impact of the COVID-19 pandemic on older adults: rapid review. JMIR Aging 4, e26474. 10.2196/26474 33720839 PMC8043147

[B9] Lind M. Bluck S. McAdams D. P. (2021). More vulnerable? The life story approach highlights older people's potential for strength during the pandemic. J. Gerontol. 76, e45–e48. 10.1093/geronb/gbaa105 32697834 PMC7454911

[B10] Maggi G. Baldassarre I. Barbaro A. Cavallo N. D. Cropano M. Nappo R.. (2021). Mental health status of Italian elderly subjects during and after quarantine for the COVID-19 pandemic: a cross-sectional and longitudinal study. Psychogeriatrics 21, 540–551. 10.1111/psyg.12703 33955115 PMC8242477

[B11] Maggi G. Baldassarre I. Barbaro A. Cavallo N. D. Cropano M. Nappo R.. (2022). Age- and gender-related differences in the evolution of psychological and cognitive status after the lockdown for the COVID-19 outbreak: a follow-up study. Neurol. Sci. 43, 1521–1532. 10.1007/s10072-021-05768-0 34820746 PMC8612768

[B12] Pearman A. Hughes M. L. Smith E. L. Neupert S. D. (2021). Age differences in risk and resilience factors in COVID-19-related stress. J. Gerontol. 76, e38–e44. 10.1093/geronb/gbaa120 32745198 PMC7454933

[B13] Rapisarda F. Vallarino M. Rosi A. Florkin A. L. Ceccato I. Lecce S.. (2023). Older adults' subjective experiences of the COVID-19 outbreak and lockdown in Italy: a qualitative study. Aging Mental Health 27, 580–587. 10.1080/13607863.2022.2087208 35723544

[B14] Santangelo G. Baldassarre I. Barbaro A. Cavallo N. D. Cropano M. Maggi G.. (2021). Subjective cognitive failures and their psychological correlates in a large Italian sample during quarantine/self-isolation for COVID-19. Neurol. Sci. 42, 2625–2635. 10.1007/s10072-021-05268-1 33914195 PMC8082482

[B15] Tsapanou A. Vlachos G. S. Cosentino S. Gu Y. Manly J. J. Brickman A. M.. (2019). Sleep and subjective cognitive decline in cognitively healthy elderly: results from two cohorts. J. Sleep Res. 28, e12759. 10.1111/jsr.12759 30251362 PMC6688963

[B16] Weil J. Kamber T. Glazebrook A. Giorgi M. Ziegler K. (2021). Digital inclusion of older adults during COVID-19: lessons from a case study of older adults technology services (OATS). J. Gerontol. Soc. Work 64, 643–655. 10.1080/01634372.2021.1919274 33882782

[B17] Wennberg A. M. V. Wu M. N. Rosenberg P. B. Spira A. P. (2017). Sleep disturbance, cognitive decline, and dementia: a review. Semin. Neurol. 37, 395–406. 10.1055/s-0037-1604351 28837986 PMC5910033

[B18] Yang Y. Li W. Zhang Q. Zhang L. Cheung T. Xiang Y. T. (2020). Mental health services for older adults in China during the COVID-19 outbreak. Lancet Psychiat. 7, e19. 10.1016/S2215-0366(20)30079-1 32085843 PMC7128970

[B19] Zhong B. L. Zhou D. Y. He M. F. Li Y. Li W. T. Ng C. H.. (2020). Mental health problems, needs, and service use among people living within and outside Wuhan during the COVID-19 epidemic in China. Ann. Transl. Med. 8, 1392. 10.21037/atm-20-4145 33313137 PMC7723535

